# Psychological impact on first responders dispatched to out-of-hospital cardiac arrest via smartphone alerting system: A longitudinal survey-based study

**DOI:** 10.1016/j.resplu.2025.100941

**Published:** 2025-03-22

**Authors:** Julian Ganter, Ariane-Catherina Ruf, Stefan Bushuven, Ute Nowotny-Behrens, Michael Patick Müller, Hans-Jörg Busch

**Affiliations:** a Department of Anaesthesiology and Critical Care, Medical Center – University of Freiburg, Faculty of Medicine, University of Freiburg., Germany; b Department of Emergency Medicine, Faculty of Medicine, University Hospital of Freiburg, University of Freiburg, Germany; c Institute for Infection Control and Infection Prevention, Hegau-Jugendwerk Gailingen, Health Care Association District of Constance, Germany; d Institute for Anesthesiology, Intensive Care, Emergency Medicine, and Pain Therapy, Hegau Bodensee Hospital Singen, Germany; e Department of Psychiatry and Psychotherapy, Medical Center – University of Freiburg, Faculty of Medicine, University of Freiburg, Germany; f Department of Anaesthesiology, Intensive Care and Emergency Medicine, St. Josefs Hospital, Freiburg, Germany

**Keywords:** First responder, Smartphone alerting systems, Out-of-hospital cardiac arrest, Automated external defibrillator, Public access defibrillation, Second victim

## Abstract

**Background:**

Smartphone alerting systems designed to dispatch volunteer first responders to out-of-hospital cardiac arrest cases are progressing rapidly. Recently, growing attention has been given to understanding the impact of these operations on first responders, with a particular focus on safeguarding them from possible psychological challenges. This study investigates the psychological stress experienced by first responders following their involvement in an operation, analyzing specific stress factors to enhance opportunities for psychological support.

**Methods:**

*A* two-stage questionnaire (Q1 and Q2) survey was conducted, with surveys administered one and four weeks after dispatched first responder arrived at the scene between October 9, 2023, and January 23, 2024. Screening questions based on the FAUST study assessed psychological stress, with ≥4 positive responses indicating at-risk individuals. Personal and operational variables were analyzed for their correlation with stress levels for identifying affected first responder. The study was approved by the Freiburg Ethics Committee (DRKS00032958).

**Results:**

The response rates for the triggered questionnaires were 190/324 (59%) for Q1 and 132/322 (41%) for Q2. Fewer than 1% answered ≥4 screening questions positively, indicating a low measured prevalence of psychological stress. Situations involving resuscitation or already deceased patients but also first responders’ feelings of insecurity were identified as significant factors of possible psychological stress, while regular CPR training appeared to significantly reduce the likelihood of stress.

**Conclusions:**

First responders who volunteer for such roles frequently encounter challenging situations. However, psychological stress is rarely reported. Integrating mechanisms into smartphone alerting systems to identify stress indicators and provide accessible support is essential.

## Background

Smartphone alerting systems that activate first responders (FR) in cases of out-of-hospital cardiac arrest (OHCA) have been in successful use for several years, and their implementation is currently endorsed by guidelines from the European Resuscitation Council (ERC) and the American Heart Association (AHA).^1^, ^2^ With advancements in technology, these systems enable FR to be geo-referenced and alerted via smartphone simultaneously with emergency medical services (EMS) dispatch^3^. Recently, attention has been focused on how smartphone alerting system (SAS) operations may affect volunteer FR and how to safeguard them from negative psychological effects.^4^, ^5^, ^6^ Research into mental health following involvement in resuscitation efforts generally indicates low levels of psychological stress.^7^, ^8^, ^9^ Nevertheless, it is crucial to identify and understand triggers and to provide support for volunteers if they show signs of stress reactions or “second victim” phenomena, which may lead to trauma and subsequent psychic disease.^10^

Indeed, a scientific statement from the AHA has called for expanded research on this subject, highlighting the current lack of recommendations or guidelines for supporting bystanders.^5^ Differences among SAS systems in terms of FR training are also noteworthy.^11^ While some systems alert laypersons, other systems are designed for professionally trained FR only. Such differences in training levels may be especially relevant when considering the psychological impact on responders.^12^ For instance, within the SAS “Region of Lifesavers”, only medically qualified volunteers are authorized, with some even possessing preclinical work experience.^13^

In this study, the psychological stress experienced by FR after responding to an operation was assessed, with an analysis of specific aspects of psychological stress conducted to enhance the provision of psychological support for FR.

## Methods

### Study design and setting

**Fig. 1:**
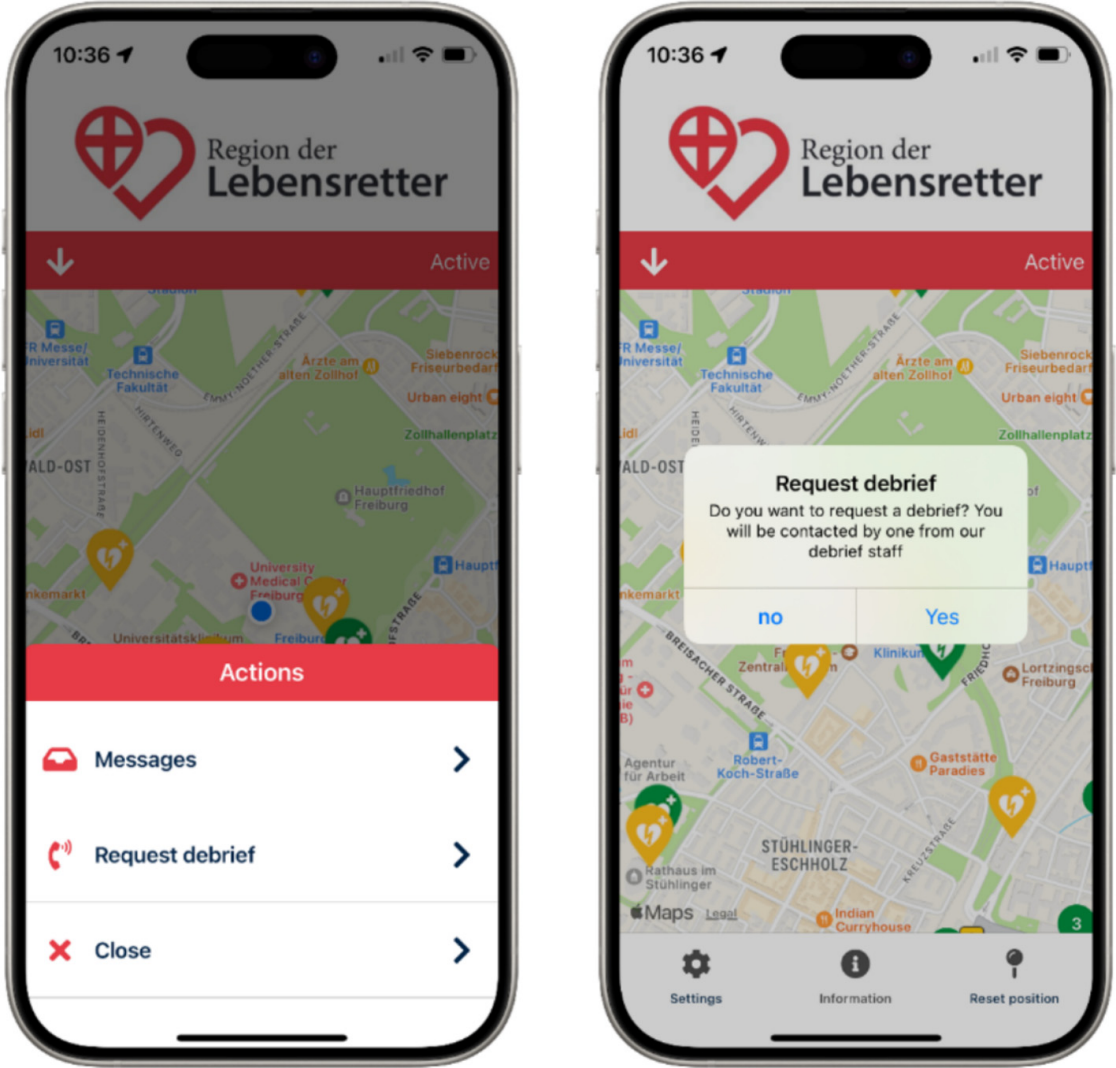
Display of the smartphone app ‘Region of Lifesavers’ in non-alarm mode. A debriefing can be requested via the menu field (left image), which must be confirmed again (right image) so that the regional managers are notified.

**Fig. 2:**
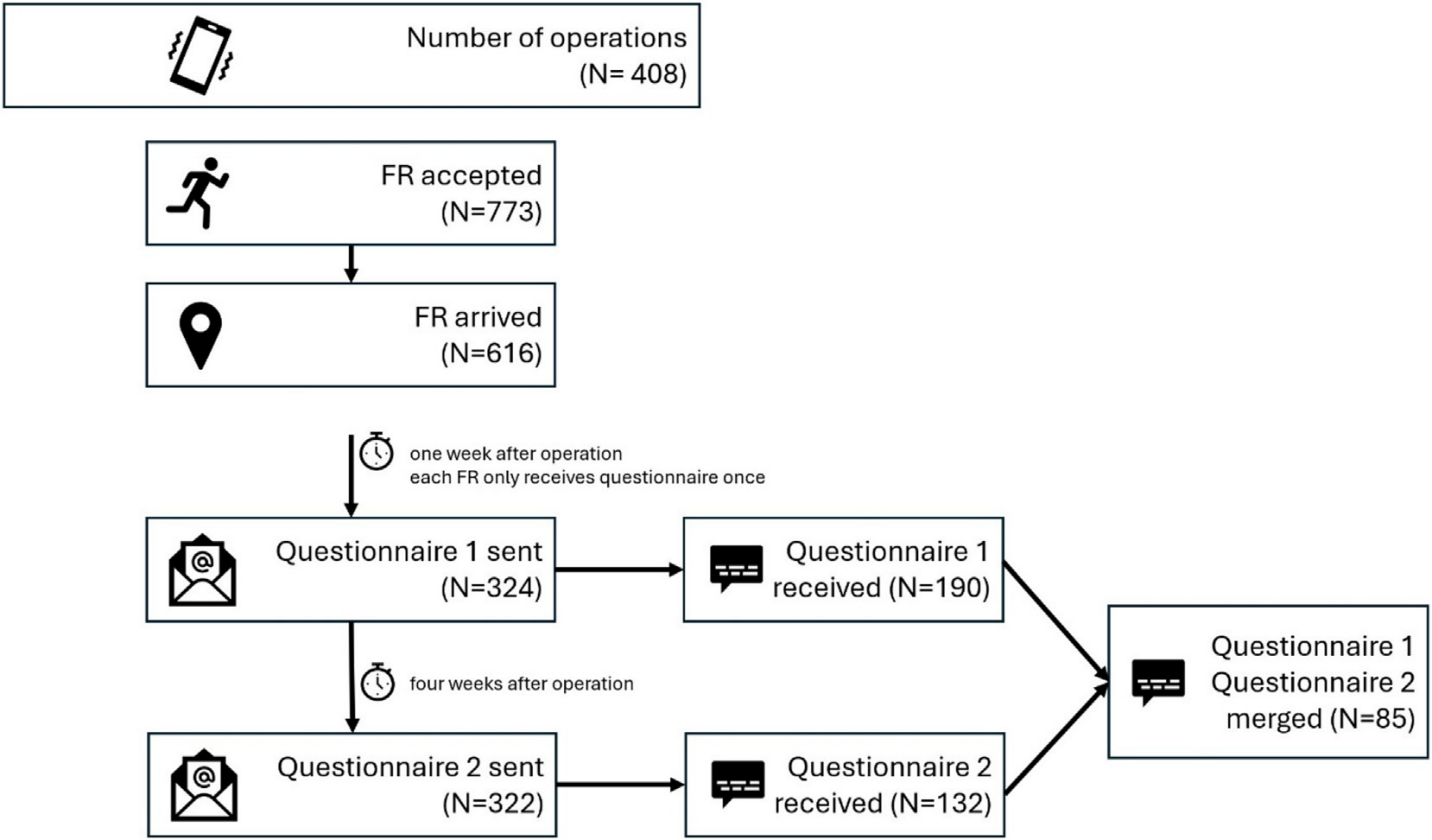
Study flow chart. A maximum of 4 first responders (FR) are assigned to an operation. Each individual FR receives both questionnaires only once during the study period. Q1 one week and Q2 four weeks after the operation.

A two-phase questionnaire survey was conducted to investigate the psychological responses and symptoms of FR who arrived on the scene of a SAS operation and put into context by a retrospective evaluation of the system. The SAS Region of Lifesavers was implemented in 2018 in the study region Freiburg/Germany (1,531 km^2^, 493,036 inhabitants).^14^ When the dispatch centre receives an emergency call and alert EMS with the keywords “unconscious” and “resuscitation”, the SAS is automatically activated, unless the dispatchers stop the operation (e.g. due to dangerous situations or threat). The system is not activated for locations in care and healthcare facilities. As many FR often receive a pre-alarm and report back their availability, there is a need to precisely select only those FR who could reach the scene of the emergency as quickly as possible. Upon activation of the system, which includes the transmission of the emergency location's geospatial coordinates and the estimated arrival time of the first rescue vehicle from the dispatch centre, the alarm server evaluates the positions of registered FR. Following a pre-alert, FR respond with their availability status (acceptance or rejection), their mode of transportation, and whether they have an automated external defibrillator (AED) on hand. The system then calculates the optimal route to the emergency scene for each FR, taking into account their personal mode of transport and the current traffic conditions. The first two FR expected to arrive at the scene are directed there immediately, where they can perform basic life support. If no FR has indicated possession of an AED, the third FR is directed to the nearest publicly accessible defibrillator (PAD). Meanwhile, the fourth FR either assists the EMS at the scene or attends to relatives. Once the operation concludes, a brief report is generated within the app for the FR to complete. Regardless of the alerting procedure, FR can request a debriefing or psychological support via the app at any time (Fig. 1). The FR-report is transferred to the system’s backend where the feedback is used for research and quality management. An automatic mailing feature was set up for the study. When the system registered a geofence-based arrival of the FR with 50 m around the scene, the FR received a link to an anonymous survey at intervals of one and four weeks (Fig. 2). Each FR received the survey link only once during the study period for the first operation. The operations took place between 09th October 2023 and 23rd January 2024.

### First responders

People with medical training are being recruited to register as FR via the app. This requires at least a certified first responder qualification (or equivalent), training in a healthcare profession and regular participation in resuscitation training. The required qualification certificates can be uploaded directly and will be verified by the regional managers. FR receive a kit with personal protective equipment (PPE) and medical equipment (bag valve mask). FR are generally covered by statutory accident insurance (SGB VII) during first aid operations. In general, FR registered in the system are covered by subsidiary liability insurance throughout Germany via the Region of Lifesavers association.

### Screening questions

The “screening questions” utilized in this study were derived from a questionnaire developed by the University Medical Center Freiburg for the FAUST study.^15^, ^16^ This questionnaire is used to identify individuals at risk for psychological issues after traumatic events. In this study, it is employed as a screening tool to identify individuals at risk for PTSD or other mental health conditions, with a score of ≥4 indicating potential risk. It is important to note that the questionnaire is not intended for diagnostic purposes but rather to facilitate early identification, ensuring individuals receive appropriate access to diagnostic services. Access to these questions is available in the supplemental material.

### Survey and study participants

An anonymous survey was conducted using the online survey platform *SoSci Survey* (SoSci Survey GmbH, Germany). Two questionnaires (Supplement) were disseminated via e-mail to FR who are registered in the region of Freiburg, participated in an operation and were documented as arrived (Fig. 2). Questionnaire 1 (Q1) was distributed after a one-week interval, while Questionnaire 2 (Q2) was disseminated four weeks after their participation in the operation to the same FR. Q1 comprises a total of 28 items. Question 1 poses the question of whether the FR has the capacity to recall the event either one week after the incident or four weeks after the incident. If the response to the question is “No,” the questionnaire is closed and the survey is terminated for the participant. Questions 2 to 10 collect personal information about the FR, while questions 11 to 16 gather incident-related information. Questions 17 to 26 represent the screening questions. Finally, questions 27 and 28 aim to evaluate the possibilities of aftercare more precisely. The Q2 comprises 17 items, with questions one and two mirroring those from the Q1 and questions 8 to 17 based on Q1 including the screening questions.

### Ethical approval

The Ethics Committee of the University of Freiburg (Germany) granted permission for the study involving human participants (23-1420, 29/08/2023). Written informed consent for participation was not required for this study according to national legislation and the institutional requirements. The SAS “Region of Lifesavers” evaluation was approved separately (23-1450-S1). The trial was registered with the German Clinical Trials Register (No: DRKS00032958).

### Data collection and statistical analysis

The operational data of the SAS Region of Lifesavers were exported from backend system and pseudonymised for analysis. The data collection for the survey study was conducted independently and collected by the SoSci Survey platform anonymously. The data were analysed and exported to MS Excel (Microsoft, Version 16.89) using parameters of descriptive statistics with SPSS (IBM Corp., Armonk, NY, USA, 29.0.2.0).

A logistic regression analysis was performed to identify influential features with an increased occurrence of positive screening questions, and thus indicating psychological stress. Python (version 3.11.7) was used, followed by the application of recursive feature elimination (RFE) using the Scikit-learn package.^17^, ^18^, ^19^ RFE is a method designed to identify the most critical variables within a dataset by iteratively training a model, ranking the importance of variables, removing the least significant one, and repeating this process until only the most relevant variables remain (Supplement). Independent variables were evaluated as potential predictors for the binary outcome (positive if at least one screening question was answered with ‘yes’). The variables examined more closely in our study included gender, age, qualifications, work experience, access to regular CPR training, frequency of operations, whether the participants have ever had a burdensome operation, any pre-existing mental illness, location of operation, arriving on-scene and the event when arrived, whether it was a special emergency situation (e.g. paediatric, suicide, crime), the FR’s insecurity in action and whether any conflict on-scene was experienced. To further refine the analysis and mitigate the risk of Type I errors, the Benjamini-Hochberg correction was applied. The Benjamini-Hochberg-Correction is a statistical method used to control the false discovery rate (FDR) when multiple hypotheses are tested and allows a balance between identifying significant results and minimizing the likelihood of false positives. The significance of the predictors was assessed using p-values, with a threshold for statistical significance set at p < 0.05.

## Results

Of the 324 questionnaires Q1 sent out (after one week), 190 (59%) were completed and of the 322 questionnaires Q2 sent out (to the same first responders after four weeks), 132 (41%) were completed (Fig. 2).

**Fig. 3:**
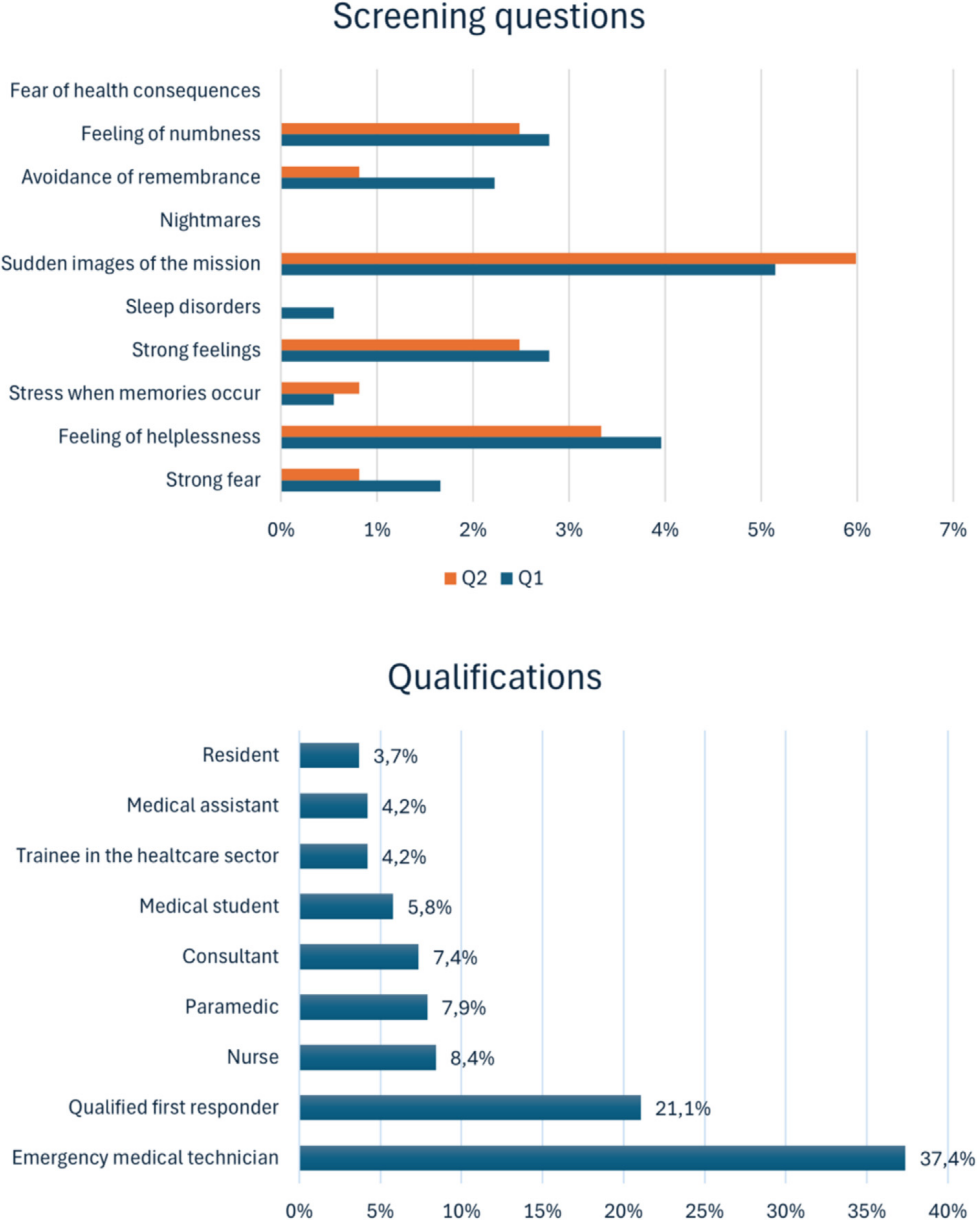
The screening questions with positive responses and the qualifications of the FR based on Table 1. The upper figure illustrates the proportion of positively answered screening questions from Questionnaires 1 and 2. The lower figure depicts the proportion of indicated qualifications for each FR, with only one response allowed per participant. The qualifications were divided into three ordinal categories: low professional qualification (trainee in the healthcare sector, medical student, qualified first responder), medium professional qualification (emergency medical technician, nurse, medical assistant) and high professional qualification (paramedic, resident, consultant).

Based on the screening questions from the FAUST study, which assess psychological stress with ≥4 positive responses indicating at-risk individuals, the results showed that in Questionnaire 1, one out of 190 participants (0.5%) answered at least four questions positively. In Questionnaire 2, none of the 132 participants provided at least four positive responses. As shown in Fig. 3, the comprehensive list includes all screening questions that were answered affirmatively and the corresponding FR qualifications.

The respective results of Q1, Q2 and the combined results when both questionnaires were completed and could be merged, are shown in Table 1.

**Table 1:** Comprehensive responses to all included questionnaires. Questionnaire 1 (Q1) was distributed one week following the operation and contains in-depth questions. Questionnaire 2 (Q2) was sent out four weeks after the operation to the same first responder and primarily focused on screening questions. The results from the combination of both questionnaires (where applicable) are presented in a separate column labeled “Merged (M)”. *NA = not applicable, SD = standard deviation, CPR = cardiopulmonary resuscitation, FR = first responder, EMS = emergency medical service.*

	Questionnaire 1 (Q1)	Questionnaire 2 (Q2)		Merged (M)		
**Sent**	324 *(= 100*% *for Q1)*	322 *(= 100*% *for Q2)*		−		
**Response**	209 (64.5%)	156 (48.5%)		−		
**Valid Responses**	190 (58.6%)	132 (41.0%)		85/132 (64.4%)		
		
				85 *(= 100*% *for M)*		
**Men**	127 (66.8%)	−		44 (51.8%)		
**Women**	62 (32.6%)			40 (47.0%)		
**Divers**	1 (0.5%)			1 (1.2%)		
**Age in years (mean, SD)**	35 ± 12.0	−		36 ± 11.9		
**Trainee in the healthcare sector**	8 (4.2%)	−		3 (3.5%)		
**Qualified first responder**	40 (21.1%)			19 (22.4%)		
**Emergency medical technician**	71 (37.4%)			32 (37.6%)		
**Paramedic**	15 (7.9%)			6 (7.1%)		
**Medical assistant**	8 (4.2%)			3 (3.5%)		
**Nurse**	16 (8.4%)			7 (8.2%)		
**Medical student**	11 (5.8%)			5 (5.9%)		
**Resident**	7 (3.7%)			4 (4.7%)		
**Consultant**	14 (7.4%)			6 (7.1%)		
**Work experience in years (mean, SD)**	9 ± 9.0	−		8 ± 7.9		
**Regular CPR training?**						
**Yes**	163 (85.8%)	−		72 (84.7%)		
**No**	26 (13.7%)			13 (15.3%)		
**NA**	1 (0.5%)			0 (0%)		
**Frequency of operations**						
**>5 operations**	90 (47.4%)	−		41 (48.2%)		
**<5 operations**	48 (25.3%)			25 (29.4%)		
**1st operation**	51 (26.8%)			19 (22.4%)		
**NA**	1 (0.5%)			0 (0%)		
**Burdensome operations**						
**Yes**						
- **Did not ask for help**	68 (35.8%)	−		35 (41.2%)		
- **Did ask for help**	25 (13.2%)			10 (11.8%)		
**No**	97 (51.0%)			40 (47.0%)		
**Pre-existing mental illness**						
**Yes**	18 (9.5%)	−		11 (12.9%)		
**No**	164 (86.3%)			71 (83.6%)		
**NA**	8 (4.2%)			3 (3.5%)		
**Location of operation**						
- **Private space**	124 (65.3%)	−		59 (69.4%)		
- **Public space**	59 (31.1%)			25 (29.4%)		
- **workplace**	5 (2.6%)			1 (1.2%)		
**NA**	2 (1.0%)			0 (0%)		
**Arriving on-scene**						
- **as first person**	57 (30.32%)	−		28 (32.9%)		
- **after FR, before EMS**	61 (32.45%)			23 (27.1%)		
- **same time as EMS**	34 (18.09%)			19 (22.4%)		
- **after EMS**	36 (19.15%)			15 (17.6%)		
**Event when arrived (Patient)**						
- **awake and responsive**	80 (42.1%)	−		28 (32.9%)		
- **unresponsive/unconscious**	33 (17.4%)			20 (23.6%)		
- **need of CPR**	52 (27.4%)			29 (34.1%)		
- **already deceased**	19 (10%)			8 (9.4%)		
**NA**	6 (3.1%)			0 (0%)		
**Special emergency situation (e.g. paediatric, suicide, crime)**						
**Yes**	12 (6.3%)	−		9 (10.6%)		
**No**	175 (92.1%)			76 (89.4%)		
**NA**	3 (1.6%)			0 (0%)		
**Confidence in action**						
**Confident**	138 (72.6%)	−		63 (74.1%)		
**Rather confident**	37 (19.5%)			17 (20%)		
**Rather uncertain**	8 (4.2%)			5 (5.9%)		
**Uncertain**	2 (1.1%)			0 (0%)		
**NA**	5 (2.6%)			0 (0%)		
**Any conflict on-scene**						
**No**	177 (93.1%)	−		81 (95.3%)		
**Yes, with the relatives**	1 (0.5%)			1 (1.2%)		
**Yes, with the patient**	0 (0%)			0 (0%)		
**Yes, with other first responders**	6 (3.2%)			2 (2.3%)		
**Yes, with the EMS staff**	3 (1.6%)			1 (1.2%)		
**Yes, with several groups of people present**	0 (0%)			0 (0%)		
**NA**	3 (1.6%)			0 (0%)		
**Informed about the debriefing request via the app**						
- **Yes, already done this**	3 (1.6%)	−		2 (2.3%)		
- **Yes, haven’t done it yet**	166 (87.4%)			75 (88.3%)		
- **Yes, would like more offers**	3 (1.6%)			2 (2.3%)		
- **No, would like more offers**	0 (0%)			0 (0%)		
- **No, I didn’t know that yet**	10 (5.2%)			6 (7.1%)		
**NA**	8 (4.2%)			0 (0%)		
**Coping strategies done (multiple selection)**	Selected	Not selected			Selected	Not selected
- **Requested debriefing via app**	1 (0.5%)	181 (99.5%)	−	−	0 (0.0%)	85 (100%)
- **Contacted counselling centre**	4 (2.1%)	178 (97.9%)			2 (2.4%	83 (77.6%)
- **Debriefed with other FR**	65 (34.2%)	117 (65.8%)			31 (36.5%)	54 (63.5%)
- **Talked with close persons**	97 (51.1%)	85 (48.9%)			44 (51.8%)	41 (48.2%)
- **Refreshed my knowledge**	25 (13.2%)	157 (86.8%)			13 (15.3%)	72 (84.7%)
- **Taken care of my physical and well-being**	32 (16.8%)	150 (83.2%)			17 (20.0%)	68 (80.0%)
- **other**	44 (23.2%)	138 (76.8%)			19 (22.4%)	66 (77.6%)
**NA**	8 (4.2%)				0 (0%)	
**Screening questions**						
- ≥ 1 positively answered question	27 (14.2%)		17 (12.9%)		13 (15.3%)	10 (11.8%)
- ≥ 4 positively answered question	1 (0.5%)		0 (0%)		1 (1.2%)	0 (0%)
					*(based on Q1)*	*(based on Q2)*
**Screening questions (single)**	Yes	No	Yes	No	Yes	No
- **Strong fear**	3 (1.6%)	181 (98.4%)	1 (0.8%)	123 (99.2%)	1 (0.6%)	169 (99.4%)
- **Feeling of helplessness**	7 (3.7%)	177 (96.3%)	4 (3.0%)	120 (97.0%)	8 (4.7%)	162 (95.3%)
- **Stress when memories occur**	1 (0.5%)	183 (99.5%)	1 (0.8%)	123 (99.2%)	0 (0.0%)	170 (100%)
- **Strong feelings**	5 (2.6%)	179 (97.4%)	3 (2.3%)	121 (97.7%)	4 (2.3%)	166 (97.7%)
- **Sleep disorders**	1 (0.5%)	183 (99.5%)	0 (0%)	124 (100.0%)	1 (0.6%)	169 (99.4%)
- **Sudden images of the mission**	9 (4.7%)	175 (95.3%)	7 (5.3%)	117 (94.7%)	12 (7.1%)	158 (92.9%)
- **Nightmares**	0 (0%)	184(100%)	0 (0%)	124 (100.0%)	0 (0.0%)	170 (100%)
- **Avoidance of remembrance**	4 (2.1%)	180 (97.9%)	1 (0.8%)	123 (99.2%)	4 (2.3%)	166 (97.7%)
- **Feeling of numbness**	5 (2.6%)	179 (97.4%)	3 (2.3%)	121 (97.7%)	2 (1.2%)	168 (98.8%)
- **Fear of health consequences**	0 (0%)	184(100%)	0 (0%)	124 (100.0%)	0 (0.0%)	170 (100%)
**NA**	6 (3.2%)		8 (6.1%)		0 (0%)	

**Fig. 4:**
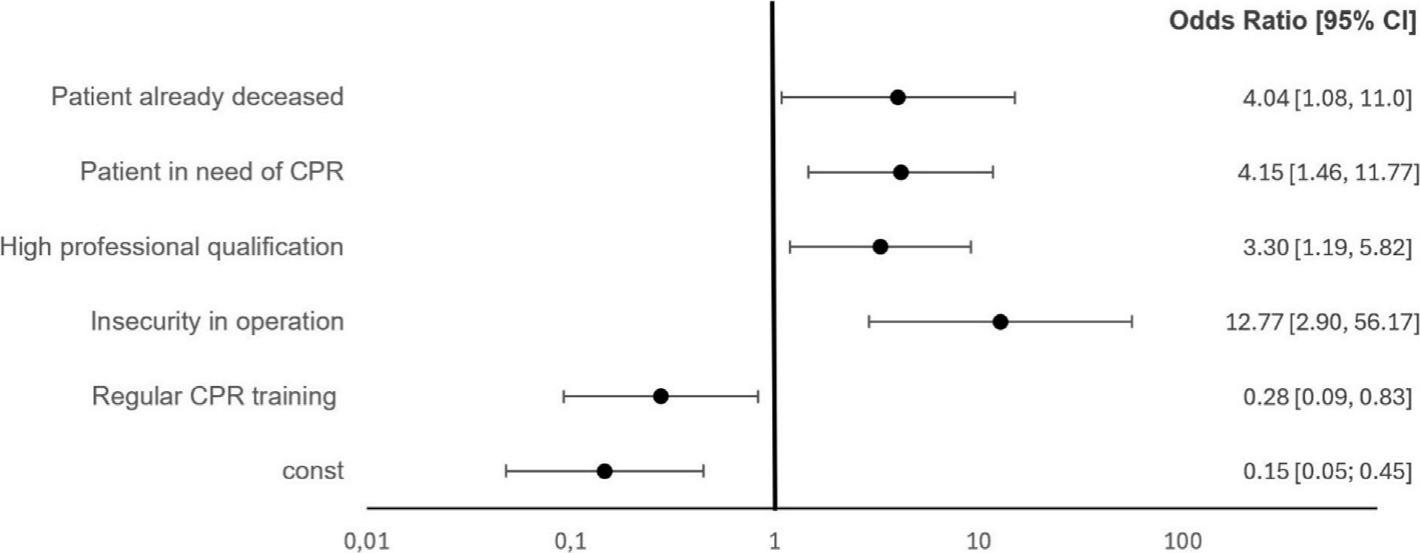
Forest plot showing odds ratios with 95% confidence intervals of the most important variables for positive screening questions (see Table 1). The vertical line represents an odds ratio of 1, indicating no effect. Variables include the patients’ status (already deceased or in need of CPR), high professional qualification, FR’s insecurity in operation and participation in regular CPR trainings. The constant represents the baseline probability of the dependent variable when all predictors are zero. It accounts for unexplained variation, ensuring accurate estimation of predictor effects.

For logistic regression 183 complete data sets (six not applicable excluded, only included gender m/f) were analysed on a positive result of the screening questions (at least one question answered with yes). After having applied RFE, a significant correlation was found for five features: “Patient in need of CPR” (p = 0.015), “Regular CPR training” (p = 0.026), “High professional qualification” (Consultant, Resident, Paramedic) (p = 0,026), “Insecurity in operation” (p = 0.002) and “Patient already deceased” (p = 0.038) (see Fig. 4).

### FR-report

After each operation, it is possible for the FR to complete a short report, which can be used for quality assessment and research purposes. Of all 773 participating FR (accepted incoming alert), 648 (84%) FR reports were received in the backend. Of the 648 FR, 405 (63%) stated that they had arrived before the EMS and 237 (37%) after the EMS. The information is missing for 6 (<1%) reports. A total of 194 comments were documented via the free text field. A keyword search using the terms ‘psych’ and ‘help’ revealed no indication of psychological signs of stress or a request for help. The comments mainly related to information on the operation process or the technical function of the app.

### Request for debriefing

During the observation period, one request for debriefing was received via the in-app debriefing feature (see Fig. 1).

## Discussion

The issue of psychological stress in FR operations is becoming increasingly significant and has already been addressed in several systems.^6^, ^20^, ^21^ The present longitudinal operation-based survey was conducted with the objective of establishing a status that could serve as a foundation for the subsequent adaptation of aftercare for FR. The survey results indicate a low measured prevalence of psychological stress. In a similar vein, Breuer et al. also observed a low level of perceived strain among FR, with less than 3%.^20^ Nevertheless, the importance of debriefing and the necessity of constant monitoring to enable a rapid response are emphasized. The authors of the present study elected to utilize a screening survey instrument to measure signs of post-traumatic stress, adding the specified sequence of questions that had previously been used in a German-language study.^15^, ^16^ The importance of additional questions that detail the circumstances of the operation (such as whether there was a conflict with the individuals involved or whether the operation occurred in private residences) has been substantiated by findings from existing literature.^21^ Consequently, the transferability of this study to other professional SAS and the references to psychological impact may be regarded as acceptable.

In the SAS “Region of Lifesaver”, only individuals in possession of a medical qualification are permitted to register as FR.^22^ The question of whether laypersons should also participate in such systems has been a subject of debate among experts to date. The primary arguments in this debate center on the question of competence in resuscitation skills. In this study, however, the focus is on the resilience aspect, which appears to be favored by the involvement of professional FR.^8^ Healthcare professionals demonstrate superior proficiency in managing the OHCA scenario compared to bystanders, exhibiting reduced tendency to attribute blame to themselves for the outcomes of patients.^23^, ^24^, ^25^, ^26^ This study exclusively focuses on professionally trained FR and is unable to provide a comparison with lay FR. Notably, this study observed higher levels of psychological stress among more qualified healthcare professionals, particularly doctors. At this point, it is important to emphasize that this observation acts as a screening tool, providing an indication of potential stress, but should not be seen as a substitute validating or confirming an actual increase in stress levels. Doctors often have a high prevalence of PTSD and the Second Victim Phenomenon, and are not necessarily familiar with the conditions of everyday pre-hospital work, as they typically do not work in the pre-hospital setting. They may also feel particularly responsible, as they hold the highest level of qualification, which can create a greater sense of accountability.^27^, ^28^, ^29^ Meta-analyses conducted on a global scale have indicated that even trained paramedics −whose daily work involves frequent exposure to rescue and crisis situations and who are well prepared for such scenarios- exhibit higher prevalence rates of PTSD compared to the general population.^30^ The results obtained serve as a foundation for the formulation of hypotheses, with the objective of identifying potential patterns rather than drawing definitive conclusions. Future research should focus on larger study populations, clearly distinguishing occupational groups and assessing prehospital experience to gain deeper insights. A high impact on self-reported psychological stress, measured by the instrument, was detected in cases where the patient required CPR or had already died. This finding is corroborated by Kragh et al., who demonstrated an association between CPR and severe psychological impact.^7^ FR operations are known to pose a significant challenge to those involved when FR personnel are not adequately prepared and encounter patients at risk of death in their leisure time. However, it appears that the situation is particularly stressful when personnel encounter patients who require immediate CPR or who already exhibit signs of imminent death. It is imperative to systematically document these events and offer targeted support to those involved, e.g. by peer support programs and professional help.^10^, ^31^

In such circumstances, the provision of debriefing for all participants should be standard practice to address and mitigate psychological stress at an early stage, thereby reducing the risk of long-term psychological consequences.^32^ However, a graduated and individually adapted approach is recommended when arranging an individual debriefing.^33^ The Region of Lifesavers app therefore offers the possibility of low-threshold support in the event of psychological stress by implementing a debriefing feature that can be used at any time, regardless of whether there is an active alarm or not (Fig. 1).

The investigation revealed that the FRs' perceived insecurity during operations was a contributing factor to their psychological distress. The study found a correlation between regular CPR training and a decrease in psychological stress levels among the FR. This finding underscores the importance of encouraging those responsible for SAS to enhance educational programs, ongoing training opportunities, and resources for registered FR. The findings highlight the significance of ongoing training in fostering a sense of competence, enhancing patient safety, and facilitating meaningful dialogue among peers. This, in turn, strengthens collaborative skills and resilience in emergency situations.^34^

As part of the system’s continued development, a debriefing request feature was implemented that allows FR to request debriefing or psychological support. This option is accessible within the app at any time, irrespective of whether an operation is currently in progress. Consequently, it can also be utilized weeks after an operation, when signs of stress may manifest with a delay. Prior to the implementation of this feature, there was only an operation-related option when the FR questionnaire was completed postoperatively.^14^ Nevertheless, it is imperative to ensure the availability of a low-threshold offer of assistance to FR, with the objective of facilitating the identification of the necessity for psychological support at the onset of the process. This approach is predicated on the recognition that individuals engaged in such operations are prone to encountering stress-inducing situations.

## Limitations

The present study examines general findings on psychological stress in a design based on voluntary information provided by the participants. The authors deliberately decided to include all FR who arrived at the scene, regardless of whether there was any contact with the patient and regardless of whether an OHCA was indeed present, to be able to identify stressful situations that are generally triggered by an operation.

The authors deemed it imperative to conduct the survey anonymously to mitigate any potential hesitation. Nevertheless, the potential for a non-responder bias remains a concern, as instances of overstraining reactions might have been underreported or may have only been consciously disclosed when participants were explicitly asked about them. The possibility of selection bias was considered, in which subjects who have experienced significant trauma might choose to decline participation. The use of an anonymous study design precluded the integration of operational data with participant responses, thus impeding the subsequent undertaking of further analysis.

## Conclusions

First responders who are dispatched using smartphone alerting systems are often called out of their leisure time to respond to stressful incidents. There are indications of psychological distress even among professional-qualified FR. Resuscitation situations, deceased patients, and the uncertainty experienced by FR in their actions are particularly stressful for volunteers. However, regular CPR training appears to mitigate the risk of psychological stress. Consequently, it is imperative that SAS systems are capable of recognizing signs of stress among FR and providing accessible support.

## Language check

During the preparation of this work the authors used DeepL SE write and OpenAI ChatGPT to check language and readability. After using this service, the authors reviewed and edited the content as needed and take full responsibility for the content of the publication.

## CRediT authorship contribution statement

**Julian Ganter:** Writing – original draft, Visualization, Validation, Supervision, Project administration, Methodology, Funding acquisition, Conceptualization. **Ariane-Catherina Ruf:** Writing – review & editing, Software, Methodology, Investigation, Formal analysis, Data curation, Conceptualization. **Stefan Bushuven:** Writing – review & editing, Validation. **Ute Nowotny-Behrens:** Writing – review & editing, Validation. **Michael Patick Müller:** Writing – review & editing, Supervision, Funding acquisition. **Hans-Jörg Busch:** Writing – review & editing, Validation, Supervision, Funding acquisition.

## Funding

This research did not receive any specific grant from funding agencies in the public, commercial, or non-for-profit sectors. Thanks to the transformative nationwide DEAL agreements (Germany), this article has been published as an Open Access version.

## Declaration of competing interest

The authors declare that they have no known competing financial interests or personal relationships that could have appeared to influence the work reported in this paper.
