# Supplementary material for: Psychological impact on first responders dispatched to out-of-hospital cardiac arrest via smartphone alerting system: A longitudinal survey-based study
Source: Resusc Plus. 2025 Mar 22;23:100941. doi: 10.1016/j.resplu.2025.100941 (PMC11995795; doi:10.1016/j.resplu.2025.100941)
Supplement: Supplementary Data 1 [file mmc1.docx]

# Questionnaire Part 1

Dear first responder,

We would like to find out how you are feeling today, how you dealt with the operation a week ago and what we can do in the future to provide you and other first responders with the best possible support.

The survey is anonymous, so we cannot draw any conclusions about you personally.

It takes about 5 minutes to complete.

We would like to thank you in advance for your participation and for your voluntary contribution to Region of Lifesavers!

### Can you remember your operation from exactly one week ago?

Yes  No

If you answered ‘**No’** to this question, you can end the survey at this point. Thank you for your participation!

### Please provide the following information to enable the creation of a personalised code to recognise the questionnaire

      First letter of your mother's first name

      Your mother's month of birth (enter two-digit numbers, e.g. September = 09)

      Second letter of your first name

      Your mother's birthday (enter two-digit numbers, e.g. 01, 02, ..., 10, etc.)

### What is your biological gender?

Female  Male  Diverse

### How old are you? (In years of life)

### Please specify the qualification that qualifies you to take part in ‘Region of Lifesavers’.

Trainee in the healthcare sector

Qualified first responder

Emergency medical technician (EMT)

Paramedic

Medical assistant

Nurse

Medical student

Resident

Consultant

### How many years have you been doing this job?

### Do you regularly take part in training courses on the field of ‘resuscitation’?

Yes  No

### How often have you already taken part in operations via ‘Region of Lifesavers’?

That was my first operation

< 5 operation

> 5 operation

### Independently of Region of Lifesavers, has there been an operation that you perceived as very burdensome?

Yes, but I did not ask for help

Yes, and I have also sought help because of it

No, I have not had any stressful operations so far

### Is there a pre-existing mental illness that affects you? (e.g. post-traumatic stress disorder, depression, etc.)

Yes, I am affected by:

No

I do not wish to give any further details.

### Where did the operation take place?

Public place (street, public transport, shop, etc.)

Private place (flat, house, etc.)

Workplace (company building, university building, etc.)

### When did you arrive at the scene?

I was the first on the scene

Other first responders were already at the scene, the EMS were not yet there

At the same time as the EMS

After EMS

### What event did you find when you arrived?

Patient was awake and responsive

Patient was unresponsive / unconscious

Patient was in need of resuscitation

Patient already dead (no action required)

### Was it a special emergency situation (e.g. paediatric emergency, suicide, violent crime)??

Yes  No

### How confident did you feel in the measures you took?

confident

rather confident

rather uncertain

uncertain

### Was there a conflict between you and the people present at the scene?

Yes, with the relatives

Yes, with the patient

Yes, with other first responders

Yes, with the EMS staff

Yes, with several groups of people present

No

### Were you very afraid (or possibly even scared to death) during the operation or shortly afterwards?*

Yes  No

### Did you feel helpless during the operation or shortly afterwards?*

Yes  No

### Do you feel very mentally stressed when you see or hear something that reminds you of the operation?*

Yes  No

### Do you feel waves of strong emotions in connection with the operation (agitation or discomfort)*

Yes  No

### Do you find it difficult to fall asleep or sleep through the night because images or thoughts about the operation come to mind?*

Yes  No

### Do you have sudden images of the operation in your head?*

Yes  No

### Do you have stressful dreams or nightmares about the operation?*

Yes  No

### Do you avoid thinking about the operation?*

Yes  No

### Do you have the impression that your ability to experience feelings is limited (you feel numb)?*

Yes  No

### Do you fear bad consequences for your health?*

Yes  No

### Are you informed that you can request follow-up interviews in the app after an operation?

Yes, I have already done this.

Yes, but I haven't done it yet.

Yes, but I would like more/different offers.

No, but I would like more/other offers.

No, I didn't know that yet.

### Please select which coping strategies you used after the operation

I requested a debriefing via the ‘Region of Lifesavers’ app

I contacted a support facility/counselling centre independently

I debriefed the operation with the other first responders/EMS

I was able to talk about it with someone close to me

I have refreshed my specialist knowledge (clinical picture, first aid measures, etc.)

I have consciously taken care of my physical and mental well-being

other:

When you accept an alarm, you never know what to expect. Perceptions and feelings before, during and after an operation are very individual. Our perceptions and feelings are influenced, among other things, by our level of training, individual experience and strategies for processing such experiences.

We’d like you to always feel good during your voluntary work at ‘Region of Lifesaver’.

* Items of the validated questionnaire of the FAUST study

# Questionnaire Part 2

Dear first responder,

We would like to find out how you are feeling today, how you dealt with the operation four weeks ago and what we can do in the future to provide you and other first responders with the best possible support.

The survey is anonymous, so we cannot draw any conclusions about you personally.

Participation takes about 2 minutes

Thank you in advance for your participation and for your voluntary contribution to Region of Lifesavers!

### Can you remember your operation from exactly one week ago?

Yes  No

If you answered ‘**No’** to this question, you can end the survey at this point. Thank you for your participation!

### Please provide the following information to enable the creation of a personalised code to recognise the questionnaire

      First letter of your mother's first name

      Your mother's month of birth (enter two-digit numbers, e.g. September = 09)

      Second letter of your first name

      Your mother's birthday (enter two-digit numbers, e.g. 01, 02, ..., 10, etc.)

### What is your biological gender?

Female  Male  Diverse

### How old are you? (In years of life)

### Please specify the qualification that qualifies you to take part in ‘Region of Lifesavers’.

Trainee in the healthcare sector

Qualified first responder

Emergency medical technician (EMT)

Notfallsanitäter*in/Rettungsassistent*in

Medical assistant

Nurse

Medical student

Resident

Consultant

### How many years have you been doing this job?

### Where did the operation take place?

Public place (street, public transport, shop, etc.)

Private place (flat, house, etc.)

Workplace (company building, university building, etc.)

### Were you very afraid (or possibly even scared to death) during the operation or shortly afterwards?*

Yes  No

### Did you feel helpless during the operation or shortly afterwards?*

Yes  No

### Do you feel very mentally stressed when you see or hear something that reminds you of the operation?*

Yes  No

### Do you feel waves of strong emotions in connection with the operation (agitation or discomfort)*

Yes  No

### Do you find it difficult to fall asleep or sleep through the night because images or thoughts about the operation come to mind?*

Yes  No

### Do you have sudden images of the operation in your head?*

Yes  No

### Do you have stressful dreams or nightmares about the operation?*

Yes  No

### Do you avoid thinking about the operation?*

Yes  No

### Do you have the impression that your ability to experience feelings is limited (you feel numb)?*

Yes  No

### Do you fear bad consequences for your health?*

Yes  No

When you accept an alarm, you never know what to expect. Perceptions and feelings before, during and after an operation are very individual. Our perceptions and feelings are influenced, among other things, by our level of training, individual experience and strategies for processing such experiences.

We’d like you to always feel good during your voluntary work at ‘Region of Lifesaver’.

# **German version (original version):**

# Fragebogen Teil 1

Liebe Ersthelferin, lieber Ersthelfer,
wir möchten hiermit in **Erfahrung** bringen, wie es dir heute geht, wie du mit dem Einsatz vor einer Woche umgegangen bist **und** was wir zukünftig tun können, um dich und andere Ersthelfer*innen bestmöglich zu unterstützen.

Die Umfrage ist anonym, einen Rückschluss auf deine Person zu ziehen ist ausgeschlossen.

Die Teilnahme dauert ca. 5 Minuten.

Wir bedanken uns vorab für deine Teilnahme und für dein ehrenamtliches Mitwirken bei Region der Lebensretter!

### Kannst du dich an deinen Einsatz von vor genau einer Woche erinnern?

Ja  Nein

Falls du diese Frage mit **„Nein“** beantwortet haben solltet, kannst du die Befragung an dieser Stelle beenden. Wir bedanken uns für deine Teilnahme!

### Bitte gib uns nachfolgend folgende Informationen an, um einen individuellen Code zur Wiedererkennung des Fragebogens zu ermöglichen

      Anfangsbuchstabe des Vornamens deiner Mutter

      Geburtsmonat deiner Mutter (zweistellige Ziffern angeben, z.B. September = 09)

      Zweiter Buchstabe deines Vornamens

      Geburtstag deiner Mutter (zweistellige Ziffern angeben, z.B. 01, 02, …, 10, usw.)

### Welches biologische Geschlecht hast du?

Weiblich  Männlich  Divers

### Wie alt bist du? (In Lebensjahren)

### Bitte gib die Qualifikation an, die dich für die Teilnahme an „Region der Lebensretter“ qualifiziert.

Auszubildende im Gesundheitswesen

Sanitätshelfer*in

Rettungshelfer*in/ Rettungssanitäter*in

Notfallsanitäter*in/Rettungsassistent*in

MFA

GuK

Medizinstudent*in

Arzt/Ärztin in Weiterbildung

Facharzt/Fachärztin

### Seit wie vielen Jahren übst du diese Tätigkeit aus?

### Nimmst du regelmäßig an Fortbildungen zum Thema „Wiederbelebung“ teil?

Ja  Nein

### Wie oft hast du bereits an Einsätzen über „Region der Lebensretter“ teilgenommen?

Das war mein erster Einsatz

< 5 Einsätze

> 5 Einsätze

### Gab es unabhängig von Region der Lebensretter einen Einsatz, den du als sehr belastend wahrgenommen hast?

Ja, aber ich habe keine Hilfe in Anspruch genommen

Ja, und ich habe deswegen auch Hilfe in Anspruch genommen

Nein, ich hatte bislang keine belastenden Einsätze

### Gibt es eine psychische Vorerkrankung, von der du betroffen bist? (Z.B. posttraumatische Belastungsstörungen, Depressionen, etc.)

Ja, ich bin betroffen von:

Nein

Ich möchte keine nähere Angabe dazu machen.

### Wo hat der Einsatz stattgefunden?

Öffentlicher Raum (Straße, ÖPNV, Einkaufsgeschäft, etc.)

Privater Raum (Wohnung, Haus, etc.)

Arbeitsstelle (Firmengebäude, Universitätsgebäude, etc.)

### Wann bist du am Einsatzort eingetroffen?

Ich war als Erste*r vor Ort

Weitere Ersthelfer*innen waren bereits vor Ort, der Rettungsdienst noch nicht

zeitgleich mit dem Rettungsdienst

nach dem Rettungsdienst

### Auf welches Ereignis, bist du bei deinem Eintreffen gestoßen?

Patient*in war wach und ansprechbar

Patient*in war nicht ansprechbar / bewusstlos

Patient*in war reanimationspflichtig

Patient*in bereits verstorben (keine Maßnahmen erforderlich)

### Handelte es sich um eine besondere Einsatzsituation (z.B. Kindernotfall, Suizid, Gewaltverbrechen)?

Ja  Nein

### Wie handlungssicher hast du dich in deinen durchgeführten Maßnahmen gefühlt?

sicher

eher sicher

eher unsicher

unsicher

### Gab es einen Konflikt zwischen dir und den anwesenden Personen am Einsatzort?

Ja, mit den Angehörigen

Ja, mit dem Patienten

Ja, mit anderen Ersthelfern

Ja, mit dem Rettungsdienst

Ja, mit mehreren anwesenden Personengruppen

Nein

### Hattest du während des Einsatzes oder kurz danach starke Angst (oder evtl. sogar Todesangst)?*

ja  nein

### Fühltest du dich während des Einsatzes oder kurz danach hilflos?*

ja  nein

### Fühltest du dich psychisch sehr belastet, wenn du etwas siehst oder hörst, das dich an den Einsatz erinnert?*

ja  nein

### Spürst du Wellen von starken Gefühlen in Zusammenhang mit dem Einsatz (Aufgewühltsein oder Unwohlsein)?*

ja  nein

### Fällt es dir schwer einzuschlafen oder durchzuschlafen, weil dir Bilder oder Gedanken zum Einsatz in den Sinn kommen?*

ja  nein

### Hast du plötzlich auftretende Bilder vom Einsatz in deinem Kopf?*

ja  nein

### Hast du belastende Träume oder Alpträume vom Einsatz?*

ja  nein

### Vermeidest du es über den Einsatz nachzudenken?*

ja  nein

### Hast du den Eindruck, dass deine Fähigkeit Gefühle zu erleben, eingeschränkt ist (dich abgestumpft fühlst)?*

ja  nein

### Befürchtest du schlimme Folgen für deine Gesundheit?*

ja  nein

### Ist dir bekannt, dass du nach Einsätzen Nachgespräche in der App anfordern kannst?

Ja, habe ich sogar schon gemacht.

Ja, habe ich aber noch nicht gemacht.

Ja, ich würde mir aber noch mehr/andere Angebote wünschen.

Nein, ich würde mir aber noch mehr/andere Angebote wünschen.

Nein, das wusste ich noch nicht.

### Bitte kreuze an, welche Strategien du nach dem Einsatz angewendet hast

Ich habe über die „Region der Lebensretter“-App eine Nachbesprechung angefordert

Ich habe mich selbstständig an eine Hilfseinrichtung/Beratungsstelle gewendet

Ich habe den Einsatz mit den anderen Ersthelfern/Rettungsdienst nachbesprochen

Ich habe mit einer mir nahestehenden Person darüber sprechen können

Ich habe mein Fachwissen aufgefrischt (Krankheitsbild, Erste-Hilfe Maßnahmen, etc.)

Ich habe mich bewusst um mein körperliches und psychisches Wohlbefinden gekümmert

sonstige:

Bei Annahme eines Einsatzes weiß man nie, was einen erwarten wird. Die Wahrnehmungen und Empfindungen vor, während und nach einem Einsatz sind sehr individuell. Unsere Wahrnehmungen und Empfindungen werden unter anderem durch Ausbildungsstände, individuelle Erfahrungswerte und Verarbeitungsstrategien solcher Erlebnisse beeinflusst.
Wir möchten, dass es euch bei eurer ehrenamtlichen Tätigkeit bei „Region der Lebensretter“ stets gut geht.

* Items des validierten Fragebogens der FAUST Studie

# Fragebogen Teil 2

Liebe Ersthelferin, lieber Ersthelfer,

wir möchten hiermit in Erfahrung bringen, wie es dir heute geht, wie du mit dem Einsatz vor vier Wochen umgegangen bist und was wir zukünftig tun können, um dich und andere Ersthelfer*innen bestmöglich zu unterstützen.

Die Umfrage ist anonym, einen Rückschluss auf deine Person zu ziehen ist ausgeschlossen.

Die Teilnahme dauert ca. 2 Minuten

Wir bedanken uns vorab für deine Teilnahme und für dein ehrenamtliches Mitwirken bei Region der Lebensretter!

### Kannst du dich an deinen Einsatz von vor genau vier Wochen erinnern?

Ja  Nein

Falls du diese Frage mit **„Nein“** beantwortet haben solltet, kannst du die Befragung an dieser Stelle beenden. Wir bedanken uns für deine Teilnahme!

### Bitte gib uns nachfolgend folgende Informationen an, um einen individuellen Code zur Wiedererkennung des Fragebogens zu ermöglichen

      Anfangsbuchstabe des Vornamens deiner Mutter

      Geburtsmonat deiner Mutter (zweistellige Ziffern angeben, z.B. September = 09)

      Zweiter Buchstabe deines Vornamens

      Geburtstag deiner Mutter (zweistellige Ziffern angeben, z.B. 01, 02, …, 10, usw.)

### Welches biologische Geschlecht hast du?

Weiblich  Männlich  Divers

### Wie alt bist du? (In Lebensjahren)

### Bitte gib die Qualifikation an, die dich für die Teilnahme an „Region der Lebensretter“ qualifiziert.

Auszubildende im Gesundheitswesen

Sanitätshelfer*in

Rettungshelfer*in/ Rettungssanitäter*in

Notfallsanitäter*in/Rettungsassistent*in

MFA

GuK

Medizinstudent*in

Arzt/Ärztin in Weiterbildung

Facharzt/Fachärztin

### Seit wie vielen Jahren übst du diese Tätigkeit aus?

### Wo hat der Einsatz stattgefunden?

Öffentlicher Raum (Straße, ÖPNV, Einkaufsgeschäft, etc.)

Privater Raum (Wohnung, Haus, etc.)

Arbeitsstelle (Firmengebäude, Universitätsgebäude, etc.)

### Hattest du während des Einsatzes oder kurz danach starke Angst (oder evtl. sogar Todesangst)?*

ja  nein

### Fühltest du dich während des Einsatzes oder kurz danach hilflos?*

ja  nein

### Fühltest du dich psychisch sehr belastet, wenn du etwas siehst oder hörst, das dich an den Einsatz erinnert?*

ja  nein

### Spürst du Wellen von starken Gefühlen in Zusammenhang mit dem Einsatz (Aufgewühltsein oder Unwohlsein)?*

ja  nein

### Fällt es dir schwer einzuschlafen oder durchzuschlafen, weil dir Bilder oder Gedanken zum Einsatz in den Sinn kommen?*

ja  nein

### Hast du plötzlich auftretende Bilder vom Einsatz in deinem Kopf?*

ja  nein

### Hast du belastende Träume oder Alpträume vom Einsatz?*

ja  nein

### Vermeidest du es über den Einsatz nachzudenken?*

ja  nein

### Hast du den Eindruck, dass deine Fähigkeit Gefühle zu erleben, eingeschränkt ist (dich abgestumpft fühlst)?*

ja  nein

### Befürchtest du schlimme Folgen für deine Gesundheit?*

ja  nein

Bei Annahme eines Einsatzes weiß man nie, was einen erwarten wird. Die Wahrnehmungen und Empfindungen vor, während und nach einem Einsatz sind sehr individuell. Unsere Wahrnehmungen und Empfindungen werden unter anderem durch Ausbildungsstände, individuelle Erfahrungswerte und Verarbeitungsstrategien solcher Erlebnisse beeinflusst.
Wir möchten, dass es euch bei eurer ehrenamtlichen Tätigkeit bei „Region der Lebensretter“ stets gut geht.
