# Supplementary material for: Psychological impact on first responders dispatched to out-of-hospital cardiac arrest via smartphone alerting system: A longitudinal survey-based study
Source: Resusc Plus. 2025 Mar 22;23:100941. doi: 10.1016/j.resplu.2025.100941 (PMC11995795; doi:10.1016/j.resplu.2025.100941)
Supplement: Supplementary Data 2 [file mmc2.docx]

Created on Sun Sep 8 14:59:14 2024

"""

import pandas as pd

import statsmodels.api as sm

from sklearn.feature_selection import RFE

from sklearn.linear_model import LogisticRegression

import shap

import matplotlib.pyplot as plt

import numpy as np

from statsmodels.stats.multitest import multipletests

''' Read data '''

data = pd.read_excel('Data.xlsx')

''' Remove columns that are not used as explanatory variables for logistic regression.

Convert relevant variables to mostly binary variables, apply one-hot encoding where applicable '''

# 1. Convert gender to binary values (Male -> 1, Female -> 0)

data['Gender'] = data['Gender'].map({'Männlich': 1, 'Weiblich': 0})

# 2. Age remains numerical, no change required

# 3. Convert qualification to three categories

def qualifikation_mapping(value):

if value in ['Auszubildende*r im Gesundheitswesen', 'Sanitätshelfer*in', 'Medizinstudent*in']:

return 'low'

elif value in ['Rettungshelfer*in/Rettungssanitäter*in', 'MFA', 'Gesundheits- und Krankenpfleger*in']:

return 'medium'

elif value in ['Facharzt/Fachärztin', 'Arzt/Ärztin in Weiterbildung', 'Notfallsanitäter*in/Rettungsassistent*in']:

return 'high'

data['Professional qualification'] = data['Professional qualification'].apply(qualifikation_mapping)

# One-Hot-Encoding for qualification (drop_first=True ensures that the first category 'low' is dropped)

data = pd.get_dummies(data, columns=['Professional qualification'], drop_first=False)

# Rename columns to more descriptive names

data.rename(columns={

'Professional qualification_low': 'Low professional qualification',

'Professional qualification_high': 'High professional qualification',

'Professional qualification_medium': 'Medium professional qualification'

}, inplace=True)

data.drop(columns=['Medium professional qualification'], inplace=True)

# Convert 'True' and 'False' values to 1 and 0

data[['Low professional qualification', 'High professional qualification']] = data[['Low professional qualification', 'High professional qualification']].astype(int)

# 4. Work experience remains numerical

# 5. Convert regular CPR training to binary (Yes -> 1, No -> 0)

data['Regular CPR training'] = data['Regular CPR training'].map({'Ja': 1, 'Nein': 0})

# 6. Convert frequency of operations to binary (>5 operations -> 1, otherwise 0)

data['Frequency of operations'] = data['Frequency of operations'].map({'>5 Einsätze': 1, '<5 Einsätze': 0, 'Das war mein erster Einsatz': 0})

# 7. Convert burdensome experiences to binary (No -> 0, otherwise 1)

data['Burdensome operations'] = data['Burdensome operations'].map({

'Nein, ich hatte bislang keine belastenden Einsätze': 0,

'Ja, aber ich habe keine Hilfe in Anspruch genommen': 1,

'Ja, und ich habe deswegen auch Hilfe in Anspruch genommen': 1})

# 8. Convert pre-existing illnesses to binary (Yes -> 1, otherwise 0) and drop additional column

data['Pre-existing mental illness'] = data['Pre-existing mental illness'].map({'Ja, ich bin betroffen von:': 1, 'Nein': 0, 'Ich möchte keine nähere Angabe dazu machen': 0})

# 9. Convert location of operation to binary (Private space -> 1, otherwise 0)

data['Location of operation'] = data['Location of operation'].map({

'Privater Raum (Wohnung, Haus, etc.)': 1,

'Öffentlicher Raum (Straße, ÖPNV, Einkaufsgeschäft, etc.)': 0,

'Arbeitsstelle (Firmengebäude, Universitätsgebäude, etc.)': 0})

# 10. Convert arrival on scene to binary (I was the first on scene -> 1, otherwise 0)

data['Arriving on-scene'] = data['Arriving on-scene'].map({'Ich war als Erste*r vor Ort': 1,

'Zeitgleich mit dem Rettungsdienst': 0,

'Weitere Ersthelfer*innen waren bereits vor Ort, der Rettungsdienst noch nicht': 0,

'Nach dem Rettungsdienst': 0})

# 11. Convert event into multiple binary variables

data['Patient unconscious'] = (data['Event when arrived (Patient)'] == 'Patient*in war nicht ansprechbar / bewusstlos').astype(int)

data['Patient awake and responsive'] = (data['Event when arrived (Patient)'] == 'Patient*in war wach und ansprechbar').astype(int)

data['Patient in need of CPR'] = (data['Event when arrived (Patient)'] == 'Patient*in war reanimationspflichtig').astype(int)

data['Patient already deceased'] = (data['Event when arrived (Patient)'] == 'Patient*in bereits verstorben (keine Maßnahmen erforderlich)').astype(int)

data.drop(columns=['Event when arrived (Patient)', 'Patient unconscious'], inplace=True) # Remove one column after OHE to prevent multicollinearity

# 12. Convert special emergency situation to binary (Yes -> 1, No -> 0)

data['Special emergency situation'] = data['Special emergency situation'].map({'Ja': 1, 'Nein': 0})

# 13. Convert confidence in action to binary (Uncertain or Rather uncertain -> 1, otherwise 0)

data['Insecurity in operation'] = data['Insecurity in operation'].map({'Unsicher': 1, 'Eher unsicher': 1, 'Sicher': 0, 'Eher sicher': 0})

# 14. Convert conflict on scene to binary (No -> 0, otherwise 1)

data['Any conflict on-scene'] = data['Any conflict on-scene'].map({'Nein': 0, 'Ja, mit anderen Ersthelfern': 1, 'Ja, mit dem Rettungsdienst': 1, 'Ja, mit den Angehörigen': 1})

''' Model the logistic regression for each of the questions 16-25 and use

questions 1-15 of the questionnaire as explanatory variables for each of the modeled

regressions '''

# Target variables (dependent variables), exclude 'Nightmares' and 'Fear of health consequences' since they only contain the value 0

data = data.drop(columns=['Nightmares', 'Fear of health consequences'])

# Convert target variables to binary values

target_columns = ['Strong fear', 'Feeling of helplessness',

'Stress when memories occur', 'Strong feelings', 'Sleep disorders',

'Sudden images of the mission', 'Avoidance of remembrance',

'Feeling of numbness']

for column in target_columns:

data[column] = data[column].map({'Ja': 1, 'Nein': 0})

# Create new target variable 'Behavioural Abnormalities': 1 if any target variable is 1, otherwise 0

data['Behavioural Abnormalities'] = data[target_columns].max(axis=1)

# Explanatory variables (independent variables)

X = data.drop(columns=target_columns + ['Behavioural Abnormalities']) # Exclude target variables

# Target variable (dependent variable)

y = data['Behavioural Abnormalities']

# Add constant (for the Y-intercept)

X = sm.add_constant(X)

# RFE with logistic regression as the base model

log_reg = LogisticRegression(solver='liblinear') # Logistic regression with liblinear solver

# Initialize Recursive Feature Elimination (RFE) to find the best predictors

rfe = RFE(log_reg, n_features_to_select=5) # Select 5 best predictors (this can be adjusted)

# Apply RFE to the data

rfe = rfe.fit(X, y)

# List of selected features (True = selected, False = not selected)

selected_features = X.columns[rfe.support_]

# Perform logistic regression with selected features

X_selected = X[selected_features] # Use only selected features

# Add a constant as a predictor for the Y-intercept

X_selected = sm.add_constant(X_selected)

# Initialize and fit the logit model

logit_model = sm.Logit(y, X_selected)

result = logit_model.fit()

# Print results

print(f"Results for target variable 'Behavioural Abnormalities':")

print(result.summary())

''' Apply Benjamini-Hochberg correction '''

# Extract p-values from the logistic regression results

p_values = result.pvalues

# Apply Benjamini-Hochberg correction

_, p_values_corrected, _, _ = multipletests(p_values, alpha=0.05, method='fdr_bh')

# Create a DataFrame to display the original and corrected p-values

p_values_df = pd.DataFrame({

"Original p-values": p_values,

"Corrected p-values": p_values_corrected

})

# Round the p-values to 3 decimal places

p_values_df = p_values_df.round(3)

print(p_values_df)

''' Graphical representation of the results '''

# Here, the logistic regression is recreated to train the model.

log_reg = LogisticRegression(solver='liblinear')

log_reg.fit(X[selected_features], y)

# Create a SHAP Explainer object for the trained model

explainer = shap.Explainer(log_reg, X[selected_features])

# Compute SHAP values for the entire dataset

shap_values = explainer(X[selected_features])

# Create a SHAP Summary plot

shap.summary_plot(shap_values, X[selected_features])

''' Calculate odds ratios and confidence intervals '''

odds_ratios = np.exp(result.params).round(3)

conf_int = np.exp(result.conf_int()).round(3)

conf_int.columns = ['2.5%', '97.5%']

# Create a DataFrame for the odds ratios and confidence intervals

odds_ratios_df = pd.DataFrame({

"Odds Ratio": odds_ratios,

"2.5%": conf_int["2.5%"],

"97.5%": conf_int["97.5%"]

})

print(odds_ratios_df)

# Determine x-axis limits dynamically

x_min = odds_ratios_df["2.5%"].min() * 0.8 # Adding some padding

x_max = odds_ratios_df["97.5%"].max() * 1.2 # Adding some padding

# Adjust x-axis limits to place the vertical line at x=1 in the middle

x_range = max(x_max / 1, 1 / x_min)

x_min_adjusted = 1 / x_range

x_max_adjusted = x_range

# Plotting the forest plot

fig, ax = plt.subplots(figsize=(8, len(odds_ratios_df) * 0.5))

# Error bars

ax.errorbar(odds_ratios_df["Odds Ratio"], odds_ratios_df.index,

xerr=[odds_ratios_df["Odds Ratio"] - odds_ratios_df["2.5%"], odds_ratios_df["97.5%"] - odds_ratios_df["Odds Ratio"]],

fmt='o', color='black', ecolor='gray', capsize=3)

# Add vertical line at x=1

ax.axvline(x=1, linestyle='--', color='blue')

# Set x-axis limit based on adjusted data

ax.set_xlim(x_min_adjusted, x_max_adjusted)

ax.set_xscale('log') # Set a log scale for a clearer view

# Manually set x-axis ticks and labels

ax.set_xticks([0.1, 1, 10, 100])

ax.set_xticklabels(['0.1', '1', '10', '100'])

# Invert y-axis and adjust y-ticks position

ax.set_yticks(range(len(odds_ratios_df)))

ax.set_yticklabels(odds_ratios_df.index, ha='right') # Align labels to the right

# Labels and title

ax.set_xlabel('Odds Ratio')

ax.set_title('Forest Plot of Odds Ratios with 95% Confidence Intervals')

plt.show()
